# Characterizing New Wintering Sites for Monarch Butterfly Colonies in Sierra Nevada, Mexico

**DOI:** 10.3390/insects11060384

**Published:** 2020-06-21

**Authors:** Ramiro Pérez-Miranda, Víctor Javier Arriola-Padilla, Martín Enrique Romero-Sanchez

**Affiliations:** National Center of Interdisciplinary Research on Conservation and Enhancement of Forest Ecosystems, National Institute for Forestry, Agriculture and Livestock Research, Progreso 5, Barrio de Santa Catarina, Coyoacan, Ciudad de Mexico 04010, Mexico; perez.ramiro@inifap.gob.mx (R.P.-M.); arriola.victor@inifap.gob.mx (V.J.A.-P.)

**Keywords:** oyamel fir forest, monarch butterfly, habitat, forest biometrics, climatic data

## Abstract

Every year, *Danaus plexippus* (Linnaeus, 1758) travels to hibernate in oyamel fir forests located between the limits of the states of Michoacán and Mexico in Mexico. Climate change and anthropogenic actions are diminishing oyamel fir forests in Mexico, putting pressure on the habitats of monarch butterflies. In the last decade, new colonies outside their usual range have been predicted through modeling and reported by the National Commission on Protected Areas of Mexico. The objectives of the study were to recover information on the historical and new hibernation sites, reported or modeled, from different literature sources. We also aimed to perform a bioclimatic and forest biometric characterization of new monarch butterfly colonies located in Sierra Nevada in Mexico to provide information to aid in conservation strategies for the monarch butterfly population. We conducted field trips to georeference the colonies at sites located in the Atlautla municipality in Mexico State. Climatic, topographic, and forest biometric variables were used to characterize the sites physically. It was found that the butterfly’s roosts occurred at a higher elevation than those recorded by other sources. The locations where the monarch’s colonies were established, in the east of Mexico State, provide information relevant to defining and developing policies for their conservation.

## 1. Introduction

The distribution of plants and animals in a place depends on historical, ecological, and physiological factors. It varies according to the biotic and abiotic conditions present in the geographical space. It is a function of not only the fundamental niche (abiotic conditions) of the place, but also several elements such as the environmental tolerance, the presence or absence of species (pollinators, dispersers, competitors, predators, and others), and the possibilities of dispersion over time and from relevant original areas [1]. Climate is a determining factor in the lifestyles and distribution of living beings, conditioning behavior, survival, the life cycle, reproduction, temporality, number of generations, distribution, and interspecific relationships [2,3,4].

Anthropogenic climate change (CC) is a phenomenon that is altering temperature and precipitation patterns, which is affecting natural processes around the world. The need to mitigate damage and adapt to the new environment poses challenges and opportunities for various sectors [5]. Due to global CC, between 20% and 30% of plant and animal species will face a higher risk of extinction, and a significant portion of endemic species will be extinct by 2050 [6]. Habitats located on different environmental gradients will be subject to change, and biological populations will not be able to adapt to the speed of the climate change phenomenon. Slow-growing species will be replaced by fast-growing species, which will affect the composition of biodiversity [7,8]. The increase in temperature is already causing modifications to the migration patterns of some insects. Already, some species that interact with vegetation have needed to expand their distribution as quickly as the flora does or change hosts [9].

The oyamel fir (*Abies religiosa* (Kunth) Schltdl. & Cham.) is a forest species that is native to the high mountains of Mexico and grows in well-drained soils of volcanic origin. It predominates in temperate climates with an average annual rainfall of greater than 1000 mm [10]. At high elevations, it is found in pure stands, but it is sometimes mixed with *Pinus montezumae* Lamb, *P. hartwegii* (Lindl), and *Pseudotsuga menziesii* var. *glauca* (Beissn.) Franco. At low elevations, it is mixed with *Quercus* spp., *Alnus acuminata* Kunth 1817, *Prunus serotina* subsp. *capuli* (Cav.) McVaugh (1951), and *Arbutus* spp. [11]. The geographical distribution of oyamel fir forests is dispersed and localized. In most cases, the communities occur as isolated patches. *Abies religiosa* forests are found in areas confined to hillsides that are usually protected from strong winds and intense radiation; they are also found in special microclimates formed by gullies or deep ravines. The largest forests are found in the Mexican Transverse Neovolcanic Axis, Sierra Madre del Sur, small regions of the Sierra Madre Occidental, and, to a lesser extent, in the Sierra Madre Oriental in Mexico [12]. Ecologically, it is a species of great importance since it serves as the annual hibernation tree (in central Mexico) of the monarch butterfly (*Danaus plexippus* Linnaeus, 1758) [7,13,14].

The monarch butterfly (*D. plexippus*) is one of the Lepidoptera that has attracted the attention of the scientific community due to the peculiarity of its migratory routes and hibernation sites [15,16]. Following Mexico’s climatic characteristics, the monarch butterfly has historically migrated to its oyamel fir forests because the low temperature and high relative humidity are well suited to the hibernation season [7,15]. Climate and weather play important but unpredictable roles in winter survival. Monarchs need cool weather to slow their metabolisms and extend their lipid reserves, but temperatures cannot fall too far. Mortality of monarchs has been found to occur at −8 and −15 °C [17]. Besides, storms greatly decrease monarch survival by wetting the butterflies and increasing their risk of freezing [4,18]. Therefore, forest quality is vital to monarchs. As fragmentation and degradation of the oyamel fir forests increases, the forests’ ability to create a suitable microclimate for the monarchs decreases.

In the last decade, oyamel fir forests within the Monarch Butterfly Biosphere Reserve (MBBR) have been under strong anthropogenic pressure due to illegal logging [13,16,19], pest diseases [19,20], and, recently, extreme weather factors such as torrential rains and droughts [17,21], probably caused by climate change. The climate, to which *A. religiosa* populations are adapted, is shifting. Therefore, the current forests are showing signs of decline [7,20,22]; moreover, forest cover in the core zone of the MBBR has been reduced, and this has a negative impact on the numbers of monarch butterflies that overwinter successfully [13,21,23,24,25]. Therefore, it is imperative to estimate areas that are potentially suitable for oyamel fir forests based on the new climate conditions.

Spatial modeling and niche ecological models combined with other analytical tools (e.g., GIS) have allowed current and future scenarios of the distribution of oyamel fir forests to be modeled [7,22,26]. Recently, numerous modeling methods and tools have been developed [3,27]. Therefore, the use of these alternatives accomplishes a double function. First, they provide knowledge about the potential distribution of the species to determine the richness and diversity of non-evaluated areas [28]. Second, they use these predictions to choose sites of interest as biological conservation zones [29,30]. The use of spatial modeling and niche ecological models has allowed us to predict potential areas for monarch butterfly hibernation outside of their regular distribution [7,22,26].

Although almost all monarch butterflies that overwinter in Mexico are contained within the limits of the MBBR, some overwintering monarchs have been found beyond the protected areas [14,31]. Within the past ten years, new monarch butterfly colonies have been registered outside of the regular wintering distribution, which confirms some of the predictions made about possible future distributions using spatial modeling [7,22,26]. For instance, there are reports confirming the presence of colonies of monarch butterfly in the forests of the Sierra Nevada, Mexico State [32], and in Real del Mar in Tijuana, Baja California [33]. These new overwintering sites could be related to the felling of the oyamel fir forests and climate change [34]. Therefore, it is important to characterize these reported sites to establish conservation strategies that aim to preserve the conditions that support the hibernation of monarch butterfly populations.

The objectives of the study were to recover information about the historical and new hibernation sites, reported or modeled, from different literature sources. We also aimed to perform bioclimatic and forest biometric characterization of new monarch butterfly colonies located in the Sierra Nevada of Mexico to provide information that aids conservation strategies for overwintering monarch butterflies.

## 2. Materials and Methods

### 2.1. Study Area

**Figure 1:**
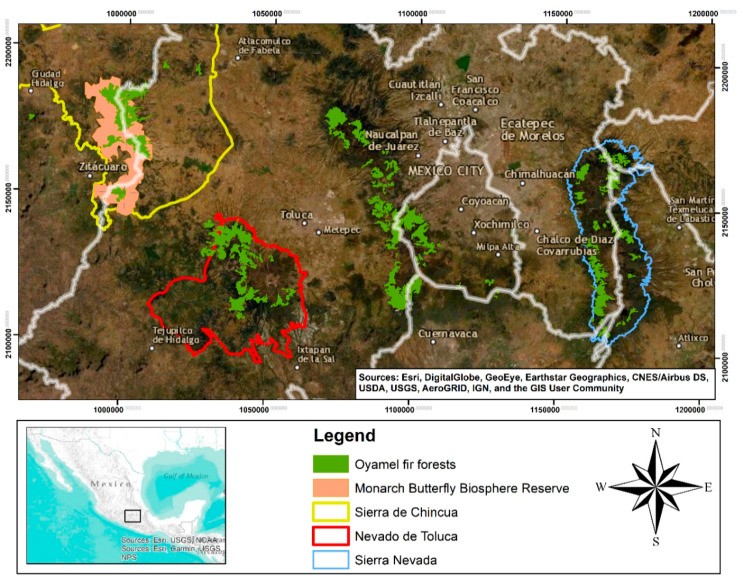
Location of the study area.

The study area was the oyamel fir forests in the central part of the Mexican Transverse Neovolcanic Axis, with a focus on the Priority Terrestrial Regions of Sierra de Chincua, Nevado de Toluca, and, notably, Sierra Nevada (Figure 1). The dominant climates in the study area are semi-cold (Cb’(w2)), i.e., sub-humid with a long cool summer, an average annual temperature between 5 and 12 °C, a temperature of between −3 and 18 °C in the coldest month, and a temperature of under 22 °C in the hottest month; temperate sub-humid (C(w1)), characterized by an average annual temperature of between 12 and 18 °C, a temperature of between −3 and 18 °C in the coldest month, a temperature of under 22 °C in the hottest month, and a precipitation-to-temperature ratio between 43.2 and 55; and temperate sub-humid (C(w2)), with the same conditions as C(w1) expect for a precipitation-to-temperature ratio that is higher than 55 [35]. The average annual rainfall varies from 558 mm in the lowlands to 900 mm in the midlands and 1708 mm in the mountains [36]. The predominant soils are Feozem, Regosol, Cambisol, Litosol, Andosol, and Fluvisol, with predominantly medium and thick textures [37].

### 2.2. Data

#### Geographic Layers

The National Institute of Statistics and Geography of Mexico (INEGI) produces and publishes land cover and vegetation type maps for the national level at a scale of 1:250,000 using a 25-hectare minimum mapping unit (INEGI 2008) [38]. From these land cover and vegetation maps, the “oyamel fir forest” land cover class was isolated by delineating the study area and providing a spatial context for the analysis. A national digital elevation model (DEM) raster dataset with a pixel size of approximately 30 m was used to create slope and aspect layers for further analysis.

Soil and topography layers at a scale of 1:250,000 were also used to extract information that was incorporated in the study later. All digital geographic data described here are freely available (www.inegi.gob.mx) and were provided by INEGI.

Monarch Butterfly Biosphere Reserve layers were provided by the National Commission of Natural Protected Areas (www.conanp.gob.mx), and the Priority Terrestrial Region layers (“Sierra Nevada”, “Sierra de Chincua”, and “Nevado de Toluca”) were available from the National Commission for the Knowledge and Conservation of Biodiversity (www.conabio.gob.mx).

Climatic data layers, such as the mean temperature and precipitation (1902–2011), were retrieved from the Digital Climatic Atlas of Mexico (DCAM). The DCAM was developed by the Informatics Unit for Atmospheric and Environmental Sciences (abbreviated to UNIATMOS in Spanish) of the National Autonomous University of Mexico (abbreviated to UNAM in Spanish).

### 2.3. Monarch Butterfly Historical Sites

Since 2004–2005, the WWF-Telmex Telcel Foundation, as part of the WWF Alliance with the Carlos Slim Foundation, has coordinated with the Directorate of the Monarch Butterfly Biosphere Reserve of the National Commission of Natural Protected Areas (CONANP) and the Ministry of Environment and Natural Resources (SEMARNAT) to systematically monitor the wintering of monarch butterflies in Mexico [14,32,39]. From the information available from the CONANP and WWF websites, we recovered the locations where colonies of monarch butterflies have historically been reported. We also reviewed scientific and non-scientific reports of sites that are not cited in the systematic monitoring system along with information describing the characteristics of the environmental conditions of each location. Additionally, we explored scientific literature reporting future predictions of the potential distribution of monarch butterfly colonies or oyamel fir forests to explain the behavior of monarch colonies across different spatial and temporal distributions.

### 2.4. Characterizing New Monarch Butterfly Wintering Sites

**Figure 2:**
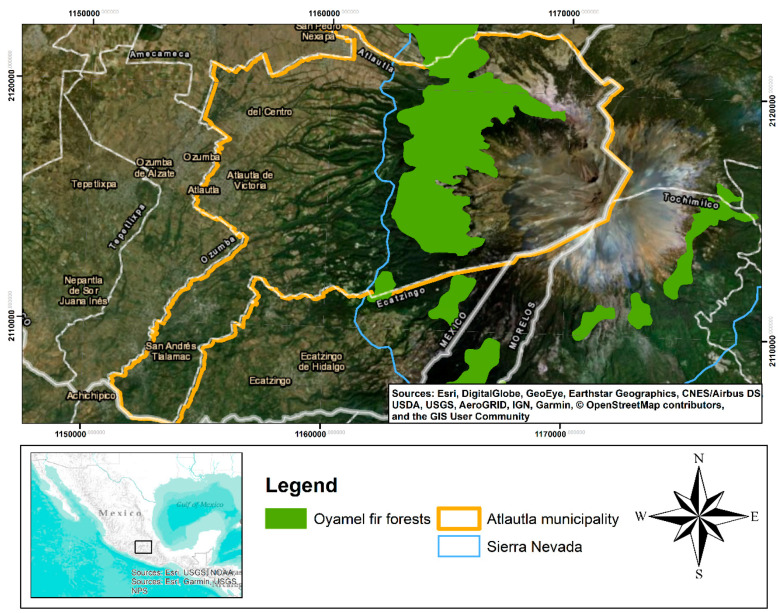
Location of the monarch butterfly in the Atlautla municipality.

Historically, wintering sites of the monarch butterfly have mainly been reported in the regions of Sierra de Chincua and Nevado de Toluca. However, a few years back, there were unofficial reports of new monarch butterfly colonies established in the municipality of Atlautla, Mexico, in the region known as Sierra Nevada (Figure 2). The National Commission of Natural Protected Areas included Atlautla as a colony site in their conservation plan [31]; however, no information about the colonies was provided, nor was any description of the environmental conditions. The first mention of the Atlautla site was in the monitoring season report for 2018–2019 [40].

For this reason, we conducted field trips to the Ejido “San Miguel Atlautla”, located in the Sierra Nevada Region, in December 2013 and 2016 and March 2017 in order to record and validate the presence of the monarch butterfly. Then, we collected georeferenced points of the colonies, extracted environmental information, and determined the status of the oyamel fir forests. To retrieve geographical data from the field, we used a global positioning system (GPS) device, the Garmin 64s. Coordinate data related to the presence of the monarch butterfly were downloaded using the program MapSource 6.13 and exported to database format (*.dbf); later, they were imported to the geographic information system (GIS) ArcMap 10.6 to generate point and polygon layers in shapefile format (*shp) for further analysis.

#### 2.4.1. Oyamel Fir Forest Conditions

To determine the status of the oyamel fir forests, we conducted a forest inventory in December 2016 and March 2017 at sites where the monarch butterfly had been detected. According to the size of the colony, forest inventory sample sites were taken. The sample plots were circular with a radius of 17.84 m (1000 m^2^), and all trees inside the site with a diameter at breast height (DBH) of greater than 7.5 cm were measured. We chose this design because it has been proven to be efficient for minimizing variance [41]. We measured the following variables: DBH, total height, forest cover, and number of trees with the presence of butterflies. The DBH was measured at 1.30 m with a 102 cm tree caliper (Haglöf; Västernorrland, Sweden), and the height was measured with a hypsometer (Vertex IV Haglöf; Västernorrland, Sweden). Additionally, we calculated the basal area for each tree and then on a plot basis according to the following equation:

$$ab=\frac{\pi }{4}\times D_{n}^{2}$$

where *ab* is the basal area in m^2^, *π* is 3.1416, and *D* is the diameter at breast height in meters.

Additionally, the coefficient of variation (CV) was calculated as follows:

$$CV=\frac{Standard\;deviation}{mean}\times \;100$$

Furthermore, we selected some sampling plots of mature stands of oyamel fir forests from the Mexican National Forest Inventory and Soils [10] close to the regions where the monarchs were detected for comparison and from the MBBR to contrast with the data taken from the Ejido San Miguel Atlautla.

#### 2.4.2. Extraction of Bioclimatic Variables

From the topographical, temperature, and precipitation layers, we extracted the values of the polygons of the colonies of the monarch butterfly to characterize the sites according to their environmental variables. For this purpose, the “Extraction” tool from the GIS “Spatial Analyst Tools” module ArcMap 10.7 was used. From each resultant file, data were exported in DBF format to be analyzed and to generate the descriptive statistics. The climate, soil, vegetation, and protected natural area variables were obtained using GIS Geoprocessing tools.

#### 2.4.3. Estimation of the Number of Butterflies at Each Site

During each visit to the hibernation colonies (December 2016 and March 2017), we delimited the perimeter using a compass and topographic tapes, taking the extreme trees occupied by butterflies as vertices of a polygon. A georeferenced tree was used as the starting point and finishing point. The area occupied by each colony was then determined using the ArcMap version 10.7 software. Later, we used the value reported in [42] of 21.1 million of butterfly/ha to make a rough estimate of the number of butterflies in “La Joya” and “Tlachanon”. The number of hectares occupied by monarchs in the overwintering area is commonly used as a proxy for population size, which is then multiplied by the density of individuals per hectare to estimate population size [42].

## 3. Results

### 3.1. Monarch Butterfly Historical Sites

**Figure 3:**
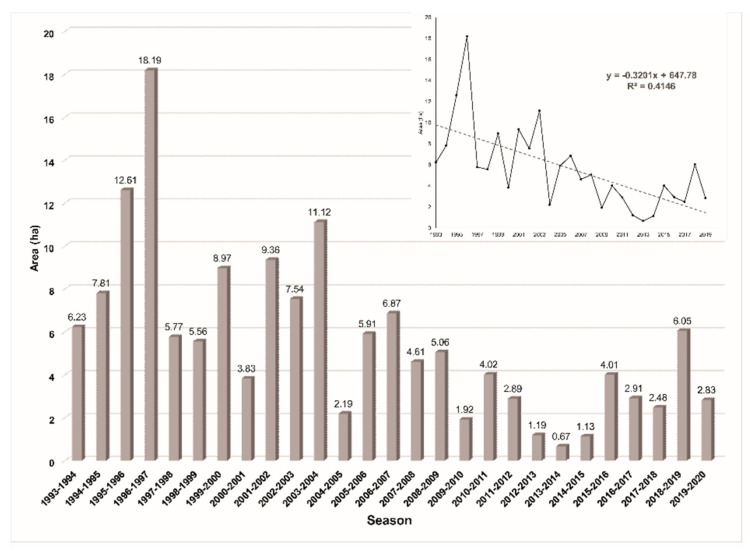
Historical trend of the area occupied by monarch butterflies (modified from Rendon-Salinas et al. [14] and Chefaoiu et al. [30]).

Since monitoring began in the winter of 1993, the forest area occupied by overwintering monarch butterflies has declined (Figure 3). The highest area recorded was in the winter of 1996–1997 with 18.19 ha, the lowest (0.67 ha) was in 2013–2014, and the 2019–2020 area was 2.83 ha [33]. The average value for the period evaluated was 5.61 ha. The data also suggest that a negative trend occurred during the period evaluated (1993–2020), as a diminished area of forest serving as hibernation sites for the monarch butterfly was found.

We assume that this tendency of decrease in the monarch occupation area can be explained by the loss of breeding habitat in the United States due to the expansion of GM herbicide-resistant crops, with consequent loss of milkweed host plants, and the reduction and degradation of forest cover within and outside the MBBR [24,43]. The main factors acting as drivers of forest cover loss are illegal logging [13,16,19], bark beetle (*Scolytus mundus* Wood, 1968, and *Pseudohylesinus* spp.) outbreaks [19,20], and extreme weather factors and climate change [7,17,21]. Table 1 presents the colonies that have historically been reported as wintering sites inside the MBBR.

Different future projections of areas show that suitable conditions for overwintering monarchs at the present overwintering sites will likely reduce in extent by 73–100% over the coming decades [4,17,22]. Some studies have already pointed out, using future projected scenarios, that specific areas in the Chichinautzín (Morelos), La Malinche (Tlaxcala), Ixtaccihuatl, and Popocatépetl volcanoes (Mexico State and Puebla) could have suitable environmental conditions for overwintering monarch butterflies [4,17,22]. These predictions can be validated with recent data that reports new wintering sites outside the MBBR, along with the two new colonies reported in this study (Table 2).

### 3.2. Characterizing New Monarch Butterfly Wintering Sites

**Figure 4:**
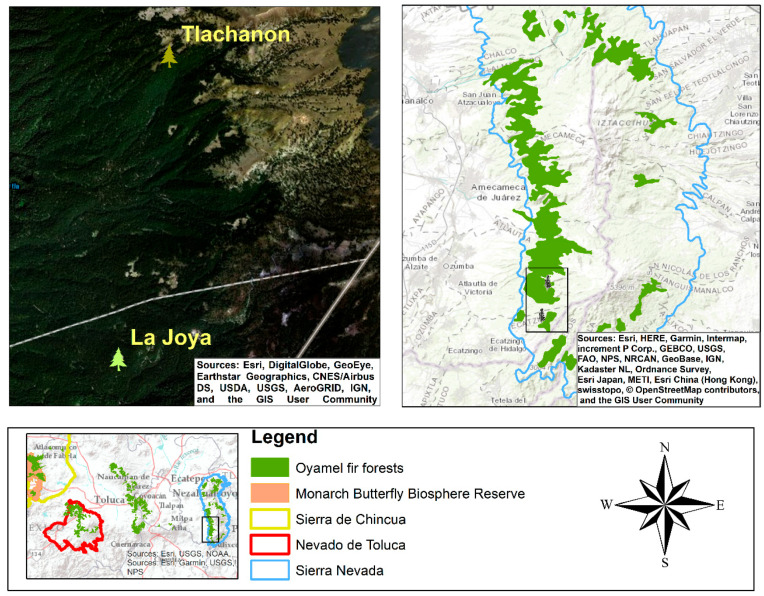
Locations of the La Joya and Tlachanon sites in the Sierra Nevada Region.

According to the field trips carried out in Sierra Nevada, we were able to identify two colonies of monarch butterflies. These two colonies were found in the Ejido San Miguel Atlautla, and the sites are known as “La Joya” and “Tlachanon”. The locations of the La Joya and Tlachanon monarch butterfly colonies were in the municipalities of Ecatzingo and Atlautla in Mexico State, respectively, approximately 5 km from the foothills of the Popocatepetl volcano and around 165 km from the MBBR (Figure 4).

**Figure 5:**
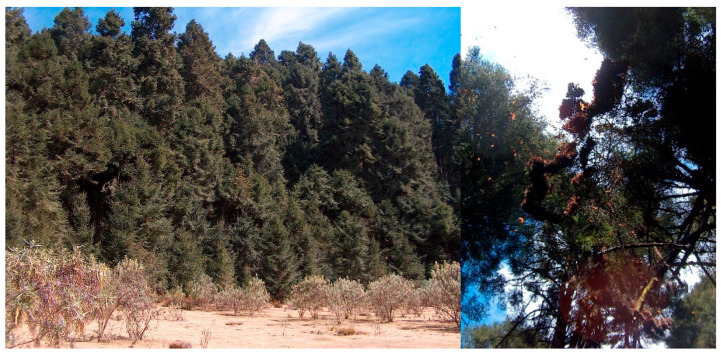
Panoramic view of the La Joya location (**left**) and clusters of monarch butterflies on an oyamel fir tree (**right**). Photos by Victor J. Arriola-Padilla.

The dominant vegetation is pure stands of oyamel fir forest. At first sight, the trees are mature, and the areas where butterflies were observed are well conserved. During the visits, we georeferenced every tree where clusters of monarch butterflies were spotted (Figure 5).

#### 3.2.1. Oyamel Fir Forest Conditions

At the La Joya site, which has full forest cover, four sample sites were established oriented to each of the cardinal directions and covering a sampled area of 4000 m^2^. An individual *A. religiosa* tree was taken as the central point. The total number of trees inventoried at the La Joya and Tlachanon sites was 125, of which 123 were firs and 2 were pines. The density of trees in La Joya was 163 individuals/hectare, and the percentage of forest cover was above 85%. Forest biometrics data measured in La Joya are described in Table 3.

Because the monarch colony at Tlachanon was established on the edge of forest and grassland, two points were selected at random, and the data were recorded in the same way as at La Joya. Meanwhile, at the Tlachanon site, the density of trees was estimated to be 300 individuals/hectare. The average forest cover was also above 80%. Forest biometric data from the Tlachanon sites are described in Table 4.

The tree densities per hectare obtained for La Joya and Tlachanon were very similar to the data recorded in systematic sampling (also known as conglomerates) for Mexico State, collected by the National Forest and Soil Inventory (INFyS). The INFyS sampling sites 61,869 in the municipality of Tlalmanalco and 62,122 in Amecameca have densities of 400 and 175 trees/ha, respectively. These values may suggest that there are potential areas for butterfly hibernation in the region.

For comparison with densities of conglomerates located in the MBBR, in values obtained for the municipalities of Sengio and Ocampo, Michoacán, and Donato Guerra in Mexico State, ranged from 463 individuals/ha at sampling site 59,564 (San Felipe del Progreso Norte) to 625 individuals/ha at sampling site 59,814 (San Felipe del Progreso Centro 1), 301 individuals/ha at sampling site 60,320 (San Felipe del Progreso Sur), and 263 individuals/ha at sampling site 60,067 (San Felipe del Progreso Centro 2). The number of trees per hectare was variable concerning those obtained at the Atlautla sites, so no definite pattern was observed. However, they were slightly denser than those found in Sierra Nevada.

The tree density per hectare for monarchs is around 375–600 individuals [44]; below this, the internal temperature of the forest diminishes, reaching the critical threshold for the survival of the insect [17]. The trees must be adults since they have a higher surface of perches and protection. Nevertheless, the density of the trees in both the La Joya” and Tlachanon sites can be considered as adequate because the tree conditions were found to create a mesoclimatic environment with little variation in its elements, which is conducive to hibernation [20].

The mean DBH values of the trees in the 61,869 and 62,122 INFyS conglomerates were 29 and 45 cm, respectively, and the total basal areas were 38.43 and 38.42.0 m^2^/ha, respectively. The values of these variables are lower than those found at La Joya and Tlachanon. Regarding conglomerates 59,564, 59,814, 60,067, and 60,320, located close to the MBBR, the DBH values were 43.1, 33.9, 30.9, and 33.3 cm, respectively, and the total basal areas were 75.37, 75.37, 21.32, and 66.05 m^2^/ha, respectively. In general, the forest biometric parameters of the La Joya and Tlachanon sites (of the new monarch butterfly colonies) were found to be relatively similar to those of the INFyS conglomerates measured close to the MBBR.

#### 3.2.2. Bioclimatic Characterization of Sites

La Joya is a concave-shaped land element with a surface area of 0.903 ha. In the lower part, it is an open and flat area with grasses and shrubby plants, and its slopes have oyamel trees. In this sampled area of the monarch butterfly colony, 26 oyamel trees with butterflies were identified. Tlachanon has a flat inclined and semi-open topography; it is a transitional zone from oyamel and pine forest to grassland with *Asclepia* sp. Five *Abies* trees with butterflies were recognized. The type of soil where the colonies “La Joya” and “Tlachanon” are located is classified as Eutric Regosol, according to the National Institute of Statistics, Geography and Information (INEGI). The topographic variables measured at each site were elevation, terrain orientation, and slope (Table 5).

The historical wintering sites of the butterflies are located between 2400 and 3100 m above sea level [31,33,39], although there have been reports that colonies can be found in elevations ranging from 2819 to 3400 m above sea level [22]. The elevations recorded in this study for La Joya and Tlachanon were between 3272 and 3538 m above sea level, the latter being the highest recorded at the time (Table 5).

Concerning exposure, the southern or southwestern slopes are preferred by the butterflies [31]. These slopes provide them with a more significant number of light hours during the day [33]. However, they can be found in other types of exposure (S, SW W, NW, N, SE, NE) [22]. The exposure recorded in this study is in accordance with that reported in previous studies. Regarding slopes, our study area was determined to be steep, 41.99° (Table 5), while other reports have mentioned less steep slopes of 23 to 26° [31] and 2 to 41° [22].

The climate predominating in both colonies above 3200 m a.s.l. is semi-cold (Cb’(w2)) sub-humid with a long cool summer, an average annual temperature between 5 and 12 °C, a temperature of between −3 and 18 °C in the coldest month, and a temperature of under 22 °C in the hottest month. The recorded climate variables are shown in Table 6.

#### 3.2.3. Estimation of the Number of Butterflies at Each Site

The estimated area occupied by butterflies in La Joya was about 0.076 ha; in Tlachanon, it was about 0.088 ha. In both sites, the value used as proxy was 21.1 million butterflies/ha [42]. The results suggested populations of 1.60 and 1.86 million of butterflies for the La Joya and Tlachanon colonies, respectively (Table 7).

## 4. Discussion

The monarch butterfly is a fascinating research organism because of its peculiar migration pattern and its unique yearly life cycle. Recent concerns about the viability and conservation of the sanctuaries in Mexico have been pointed out [39,45]. A statistically significant decline in monarch butterfly colonies has been documented [43]. According to data available from the monitoring program in Mexico of the wintering of the monarch butterfly, 19 colonies have been registered as historical hibernation sites. However, it is worth noting that the number of colonies has been variable in each season, and only three of them have been consistently occupied [14,32,40].

The variation in the number of colonies could be influenced by the composition and structure of the oyamel forests where the butterfly seeks its ecological niche due to their climatic and physiological characteristics, humidity, elevation, and exposure, which create unique environmental conditions suitable for its biology [18,20,42]. Although there is no consensus on the explanation for the variation in the number of colonies in the reports, some studies have suggested that environmental conditions are changing, which could affect the presence of the butterflies, at least in the MBBR, in the future [7,21,39,46].

Anthropogenic pressure over the biosphere reserve is causing the forest cover to diminish in a very accelerated manner. During 2001–2012, 1254 ha was deforested and 925 ha was degraded in the Monarch Butterfly Biosphere Reserve [39]. Deforestation and forest degradation in the MBBR are factors that could cause populations of monarchs to look for new hibernation sites. Another big issue that has recently gained relevance is the land-use change within the reserve and around it because of the high demand and price of avocados driving an increase in plantations. Global Forest Watch (GFW) data show that forest clearing to establish avocado plantations has been pushed into the boundaries of the MBBR. Satellite images have revealed large areas with agricultural ponds, often with traces of burning, a distinct pattern indicative of avocado expansion [47].

The existence of the new monarch butterfly colonies in Sierra Nevada could be related to the pressure suffered by their historical hibernation sites. Their establishment in different areas may be part of the seasonal migratory dynamics that existed during their journeys to their historical habitats, as indicated in the species migration map [33]. It is worth noting that the colonies at the new locations are no more than 15 years old but have been consistently occupied according to local people from the Ejido San Miguel Atlautla.

The temperature in the colonies located in the mountains of Michoacán varies from 1 °C at night and early in the morning to 13 °C in the afternoon; during cloudy and rainy or snowy periods, the daytime temperature remains at an average of 10 °C [48]. This temperature variation reported in Michoacán gives a view of a possible future scenario in Tlachanon and La Joya, where the minimum temperature in the period of hibernation reaches 0.8 °C, bringing a possibility of snowfall. During the hibernation period of the monarch butterfly, the temperature recorded in the Ejido San Miguel Atlautla had an average minimum of 0.8 °C and an average maximum of 17 °C. These values are very close to those indicated in other studies [31], which pointed out that butterflies prefer temperatures that fluctuate between 3 and 18 °C.

Given the new scenarios that would arise as a result of climate change, the ideal environmental conditions for the development of oyamel fir forests may be found at higher elevations close to the highest volcanoes in Mexico [7]. Our study may provide evidence supporting this hypothesis since the La Joya and Tlachanon sites are the first places where the monarch butterfly is migrating to in the oyamel fir forests at higher elevations.

One alternative proposed for future climatic conditions is assisted migration of fir forests [7]. However; even if assisted migration from oyamel fir forests were to occur, it would have to be carried out in a planned manner, since the transition to a new climate pattern is an unpredictable way for the monarch butterfly to find its new ecological niche. Another option is the use of ecological niche modeling to predict or identify areas where environmental conditions are suitable for monarchs. Models predicting the presence of monarchs with considerable accuracy suggest that the ecological niche of the monarch butterfly is more widely distributed than the occurrence of known colonies [4], as has been proved in this study. Therefore, future searching could locate new colonies and identify areas with suitable conditions for the species. Although species rarely inhabit the entire spatial distribution of their ecological niches [49], our suggestion that additional suitable locations could exist outside the region of known wintering sites was one motivation for this study as well as future studies.

In this sense, the combination of spatial modeling and niche ecological models with other analytical tools (e.g., geographic information system (GIS)) offers an option for evaluating non-inventoried sites and modeling past and future scenarios of the distribution of species [19,50]. Recently, numerous modeling methods and tools have been developed [3,27], but they have mainly been used in ecology and biogeography [49,51]. Therefore, the use of these alternatives accomplishes a double function. First, they provide knowledge about the potential distribution of the species to determine the richness and diversity of non-evaluated areas. Second, they can be used to predict ideal sites for biological conservation zones [29,30]. These arguments represent a path and direction for future research. Finally, the study of the quality of the habitat (cover, sources of food, space, and water) in La Joya and Tlachanon is also worth highlighting as a research path.

## 5. Conclusions

We presented and characterized two new wintering sites for the monarch butterfly. The bioclimatic characteristics of the new wintering sites are similar to those recorded historically by several authors in the MBBR. The variable that was observed to present a new range was elevation, with the one presented in this study being the highest recorded. It was also observed that the minimum temperature recorded at the new wintering sites had a variation of 0.2 °C. The forest biometric information recorded to date has been variable; however, it is considered essential to point out that the colonies occur in sites with mature and well-conserved trees. Due to the constant disturbance of the oyamel fir forests by anthropogenic causes and climate change, it should be considered essential to characterize other stands in Mexico State and Michoacán to determine more potential areas for hibernation of the monarch butterfly.

## Figures and Tables

**Table 1:** Reported sites for wintering of the monarch butterfly inside the Monarch Butterfly Biosphere Reserve (MBBR).

State	Sanctuary	Colony	Source
Michoacán	Cerro Altamirano	Ejido Contepec	[14,37]
	Chivati-Huacal	Indigenous community Carpinteros	[14,37]
	Sierra Chincua	Federal property	[14,37]
		Ejido Cerro Prieto	[14,37]
		Ejido El Calabozo	[14,37]
	Lomas de Aparicio	Indigenous community Crescencio Morales.	[14,37]
México State	Cerro Pelón	Ejido Nicolas Romero	[14,37]
		Indigenous community San Juan Xoconusco	[14,37]
		Ejido El Capulín	[14,37]
		Ejido Mesas Altas de Xoconusco	[14,37]
		Indigenous community San Pablo Malacatepec	[14,37]
	Sierra Campanario	Ejido el Rosario	[14,37]
		Ejido la Mesa	[14,37]

**Table 2:** Reported sites for wintering of the monarch butterfly outside the MBBR.

State	Sanctuary	Colony	Source
Michoacán	Los Azufres	Private Property San Ándres	[14,37,39]
	Mil Cumbres	Ejido Río de Parras	[14,37,39]
México State	Cerro del Amparo	Ejido San Francisco Oxtotilpan	[14,29,37]
	Palomas	Ejido San Antonio Albarrames	[14,37,39]
	Piedra Herrada	Ejido San Mateo Almomoloa	[14,37]
	Cerro de la Antena	Ejido El Potrero	[30]
	Peña Ahumada	Ejido Ojo de Agua	[30]
	Atlautla municipality	Ejido San Miguel Atlautla: “Tlachanon”	This study
	Ecatzingo municipality	Ejido San Miguel Atlautla: “La Joya”	[30], This study

**Table 3:** Forest biometric variables of the tree structure in the La Joya sites.

Sample Site	Number of Individuals	Average DBH (cm)	Average Height (m)	Basal Area (m^2^/0.10 ha)
1	11	78.60 (15.5)	37.18 (4.89)	5.54
2	21	47.76 (25.85)	28.04 (8.7)	4.90
3	20	48.39 (31.7)	29.45 (10.51)	5.63
4	13	64.43 (23.93)	33.07 (10.32)	4.82
Total	65	-	-	20.9
Average	-	57.3 (26.67)	31.15 (9.08)	5.22 *
CV	(%)	50.02	31.45	

SD, standard deviation; *, average basal area (m^2^/0.4 ha); DBH, diameter at breast height; CV, coefficient of variation.

**Table 4:** Forest biometric variables of the tree structure in the Tlachanon sites.

Sample Site	Number of Individuals	Average DBH (cm)	Average Height (m)	Basal Area (m^2^/0.10 ha)
1	37	39.98 (12.3)	28.9 (6.05)	5.08
2	23	42.10 (28.9)	24.4 (9.96)	4.65
Total	60	-	-	9.73
Total Average		40.79 (20.22)	27.29 (8.09)	4.86 **
CV	(%)	49.5	10.5	

Standard deviation in parentheses; **, average basal area (m^2^/0.2 ha); CV, coefficient of variation.

**Table 5:** Physiographic variables of the La Joya and Tlachanon sites.

Variables	La Joya	Tlachanon
Elevation (range)	3272 to 3354	3457 to 3538
Elevation (average)	3302	3496
Direction	Flat (0), North (259), East (90), South (180), West (270).	South (160) to West (274)
Mean direction	South (199)	Southwest (217)
Slope	90% (41.99°)	90% (41.99°)

**Table 6:** Temperature and precipitation of the La Joya and Tlachanon sites during the monarch butterfly hibernation period (November to February).

Variable Description	La Joya	Tlachanon
Minimum temperature (°C)	1.5	0.8
Mean temperature (°C)	9.3	8.3
Maximum temperature (°C)	17.0	15.7
Precipitation (mm)	157	147

**Table 7:** Rough estimate of population density of monarch butterflies at the study area.

Variable Description	Area (ha)	Population (million)
La Joya	0.076	1.60
Tlachanon	0.088	1.86
Total	0.164	3.46
